# Ritonavir’s Evolving Role: A Journey from Antiretroviral Therapy to Broader Medical Applications

**DOI:** 10.3390/curroncol31100450

**Published:** 2024-10-08

**Authors:** Mariana Pereira, Nuno Vale

**Affiliations:** 1 PerMed Research Group, Center for Health Technology and Services Research (CINTESIS), Rua Doutor Plácido da Costa, 4200-450 Porto, Portugal; 2 CINTESIS@RISE, Faculty of Medicine, University of Porto, Alameda Professor Hernâni Monteiro, 4200-319 Porto, Portugal; 3 ICBAS—School of Medicine and Biomedical Sciences, University of Porto, Rua de Jorge Viterbo Ferreira 228, 4050-313 Porto, Portugal; 4 Department of Community Medicine, Health Information and Decision (MEDCIDS), Faculty of Medicine, University of Porto, Rua Doutor Plácido da Costa, 4200-450 Porto, Portugal

**Keywords:** ritonavir, drug repurposing, translational medicine, oncology, chemosensitization

## Abstract

Ritonavir is a protease inhibitor initially developed for HIV treatment that is now used as a pharmacokinetic booster for other antiretrovirals due to it being a cytochrome P450 3A4 enzyme and P-glycoprotein inhibitor. Consequently, ritonavir is of special interest for repurposing in other diseases. It had an important role in battling the COVID-19 pandemic as a part of the developed drug Paxlovid^®^ in association with nirmatrelvir and has shown effects in hepatitis and other pathogenic diseases. Ritonavir has also shown promising results in overcoming drug resistance and enhancing the efficacy of existing chemotherapeutic agents in oncology. Evidence of cancer repurposing potential was demonstrated in cancers such as ovarian, prostate, lung, myeloma, breast, and bladder cancer, with several mechanisms of action presented. In vitro studies indicate that ritonavir alone can inhibit key pathways involved in cancer cell survival and proliferation, causing apoptosis, cell cycle arrest, endoplasmic reticulum stress, and metabolic stress due to the inhibition of molecules like heat shock protein 90 and cyclin-dependent kinases. Ritonavir also causes resistant cells to become sensitized to anticancer drugs like gemcitabine or docetaxel. These findings indicate that repurposing ritonavir, either on its own or in combination with other medications, could be a promising approach for treating various diseases. This is particularly relevant in cancer therapy, where ritonavir repurposing is the central focus of this review.

## 1. Introduction

Since its first licensing in 1996, the powerful HIV protease inhibitor ritonavir has been crucial in the treatment of HIV/AIDS [1]. Its function has changed over time from that of a standalone antiviral to that of an essential part of combination treatments, especially because of its capacity to inhibit the cytochrome P450 3A4 enzyme, which raises the effectiveness of other antiretrovirals [2]. Ritonavir’s dual function as a pharmacokinetic enhancer and therapeutic agent has broadened its use beyond the treatment of HIV, with the potential for this drug to be repurposed in the treatment of various illnesses and even some types of cancer. Despite its effectiveness, ritonavir’s intricate pharmacological interactions with different cytochrome enzymes provide substantial difficulties in clinical practice that must be carefully managed to maximize treatment results. To demonstrate the continued development of this adaptable medication, this review will examine the pharmacological profile of ritonavir, its current therapeutic uses, and the possibility of repurposing it to treat cancer and other viral diseases.

## 2. Pharmacological Profile of Ritonavir

**Figure 1:**
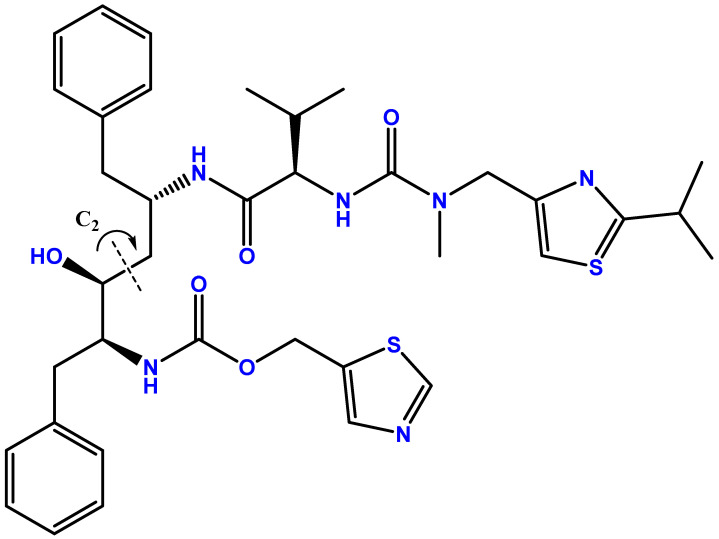
Chemical structure of ritonavir. Developed with ChemBioDraw^®^ Ultra version 13.0., a chemical drawing software. Available online: https://chemdrawdirect.perkinelmer.cloud/js/sample/index.html (accessed on 28 August 2024).

Ritonavir, an HIV protease inhibitor, is used in combination with other antivirals to treat HIV infection. Its chemical structure resembles a pseudo-C2-symmetric small molecule inhibitor [3]. Generally, C2 molecules are two-fold symmetric over a single axis when rotating the molecule 180°, but ritonavir is not fully symmetric, hence why it is called pseudo-C2-symmetric [4] (Figure 1). Initially approved in 1996, ritonavir has evolved from a standalone antiviral to a crucial component in combination therapies for HIV infections [1]. Ritonavir inhibits the viral protease enzyme in HIV-1, preventing the maturation of new viral particles and reducing viral replication. By blocking HIV-1 protease, it interferes with the cleavage of viral polyprotein precursors *gag* and *pol* into individual functional proteins required for infectious HIV-1 [5].

The absolute bioavailability of ritonavir in humans has not been determined, but after a 600 mg dose of the oral solution, peak concentrations are typically achieved in approximately 2 h under fasting conditions and 4 h under non-fasting conditions [6]. In rats, the absolute bioavailability was placed between 74% and 76.4%, depending on the use of a non-compartmental or compartmental model [7]. Its volume of distribution is 0.41 ± 0.25 L/kg, and it is primarily bound to human serum albumin and alpha-1 acid glycoprotein, with 98–99% binding over a concentration range of 0.01–30 µg/mL. The red blood cell/plasma ratio for ritonavir is 0.14. Ritonavir is extensively metabolized by the liver, primarily via the cytochrome P450 3A (CYP3A) enzyme, with CYP2D6 also contributing. Five metabolites have been identified in human urine and feces, with the major metabolite being the isopropylthiazole oxidation metabolite (M-2), which has antiviral activity like the parent drug but is found at low concentrations in plasma. After a 600 mg dose of ^14^C-ritonavir oral solution, 11.3 ± 2.8% of the dose is excreted in the urine, with 3.5 ± 1.8% as unchanged ritonavir. Additionally, 86.4 ± 2.9% of the dose is excreted in the feces, with 33.8 ± 10.8% as unchanged ritonavir. Upon multiple dosing, ritonavir accumulation is less than predicted from a single dose, possibly due to a time- and dose-related increase in clearance [6].

## 3. Current Clinical Uses

Ritonavir, initially approved for use nearly 20 years ago, has seen significant clinical application primarily as part of combination therapy for HIV treatment. Initially, high-dose ritonavir was shown to be potent in reducing plasma HIV RNA levels, using doses of 600 mg twice daily [8]. However, it was quickly learned that long-term monotherapy resulted in the emergence of mutations and HIV-1 strains resistant to ritonavir and other protease inhibitors like indinavir and saquinavir. Consequently, its use in combination with other antiretrovirals is preferred to prevent resistance development [2].

Ritonavir is extensively used as a pharmacokinetic enhancer or “booster” in HIV therapy. Lower doses significantly boost the pharmacokinetic profiles of other protease inhibitors (PIs) by inhibiting the cytochrome P450 (CYP) 3A4 enzyme, leading to higher drug concentrations, reduced pill burden, and simplified dosing regimens. This enhancement is crucial for drugs like saquinavir, atazanavir, darunavir, and others, where ritonavir increases their exposure, efficacy, and clinical effectiveness [9]. When used in combination as an enhancer for other HIV drugs, lower ritonavir doses are used, typically between 100 and 200 mg twice daily [10].

Ritonavir has significant interactions with cytochrome P450 (CYP) enzymes, both as an inhibitor and an inducer, which can impact the metabolism of various drugs. Ritonavir is a potent inhibitor of the CYP3A subfamily, particularly CYP3A4, and, although some theories have been suggested, the specific mechanisms of inhibition are not yet completely understood [11]. This irreversible inhibition is a primary mechanism for its use as a pharmacokinetic enhancer, as it increases the plasma levels of co-administered drugs that are metabolized by CYP3A4. By inhibiting CYP3A4, ritonavir reduces the metabolism of these drugs, thereby enhancing their therapeutic effects and duration [12]. In addition to its inhibitory effects, ritonavir can also induce the expression of several CYP enzymes, though to a lesser extent. These include CYP1A2, CYP2B6, CYP2C9, and CYP2C19. Ritonavir induces these enzymes through activation of the pregnane X receptor (PXR), which increases the transcription of these CYP genes. This induction can lead to increased metabolism of drugs that are substrates of these enzymes, potentially reducing their efficacy [13].

The consequences of CYP inhibition and induction by ritonavir are significant. The inhibition of CYP3A4 results in higher plasma concentrations of drugs metabolized by this enzyme, enhancing their therapeutic effects and potentially increasing the risk of adverse effects. Conversely, the induction of other CYP enzymes can lead to increased metabolism of drugs, potentially reducing their plasma levels and therapeutic efficacy. The dual role of ritonavir as an inhibitor and inducer of CYP enzymes can complicate drug–drug interactions, requiring careful management and dose adjustments of co-administered drugs. Ritonavir’s ability to both inhibit and induce various CYP enzymes necessitates careful consideration of potential drug interactions in clinical practice to ensure optimal therapeutic outcomes and minimize adverse effects [14].

**Figure 2:**
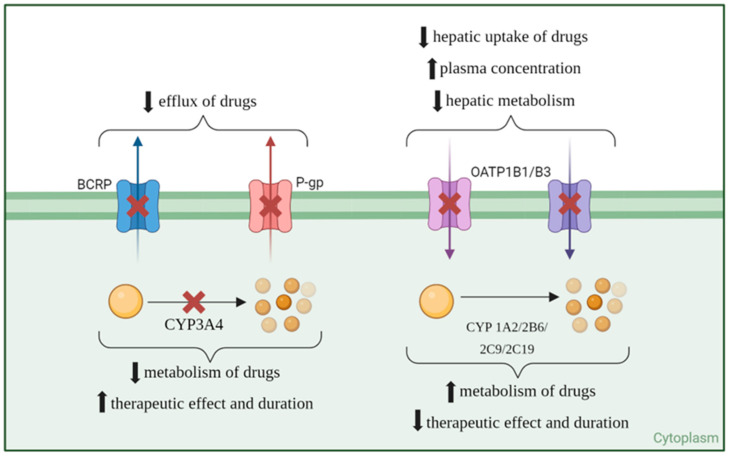
Inhibition (red cross) and induction (green upvote arrow) of transporters and metabolization enzymes of co-administered drugs (yellow ball) by ritonavir.

Apart from the effect on CYP enzymes, ritonavir has also been shown to inhibit another important class of compounds: the drug transporters. Ritonavir is known to inhibit P-gp expression and activity, which can alter the pharmacokinetics of co-administered drugs [15]. For example, ritonavir affects the distribution of indinavir and significantly increases the systemic exposure of fexofenadine, a P-gp substrate [16]. Furthermore, ritonavir acts as an inhibitor of breast cancer resistance protein (BCRP), alongside saquinavir and nelfinavir, enhancing intracellular drug concentrations by inhibiting BCRP-mediated efflux. This inhibition is clinically significant, as it can affect the bioavailability of drugs that are substrates for BCRP [17]. OATP1B1, OATP1B3, and OATP2B1 are organic anion-transporting polypeptides that play crucial roles in the hepatic uptake of various endogenous compounds and xenobiotics, including multiple drugs. Ritonavir exhibits strong inhibitory effects on both OATP1B1 and OATP1B3. Additionally, ritonavir effectively inhibits OATP2B1, although the inhibitory effects are substrate-dependent. This illustrates the complexity of ritonavir’s interaction with different OATP isoforms, emphasizing the importance of context in understanding these interactions [18]. Figure 2 shows a visual representation of ritonavir’s inhibition or induction of drug transporters and metabolizers and the consequent effect on co-administered drugs’ concentration and effect.

## 4. Potential for Repurposing for Infectious Diseases

### 4.1. COVID-19

In the context of the COVID-19 pandemic, ritonavir has been combined with nirmatrelvir to form the drug Paxlovid (Pfizer, NY, USA). Nirmatrelvir is a protease inhibitor of the SARS-CoV-2 virus’ 3CL protease, a key enzyme for viral replication [19], while ritonavir boosts its concentration to effective levels. This combination was specifically developed to target SARS-CoV-2, the virus responsible for COVID-19 [20].

Early clinical trials demonstrated that nirmatrelvir/ritonavir (NMV-r) could reduce the risk of hospitalization or death in high-risk, unvaccinated COVID-19 outpatients when administered within five days of symptom onset [21]. As a result of these positive outcomes, regulatory agencies granted emergency use authorizations (EUAs) for Paxlovid for the treatment of COVID-19 in high-risk patients with dosages of 300 mg of nirmatrelvir and 100 mg of ritonavir twice daily [22].

The World Health Organization (WHO) strongly recommended using NMV-r in patients at the highest risk of severe illness or hospitalization due to COVID-19. This includes specific guidance for its use in pregnant and lactating women. However, the WHO did not recommend NMV-r for patients with severe COVID-19, suggesting using other treatments like corticosteroids and interleukin-6 receptor blockers instead. Additionally, it was conditionally recommended for those with non-severe illnesses at the lowest risk of hospitalization [23].

Two studies on the use of NMV-r for COVID-19 treatment in vaccinated patients highlight its effectiveness. The first study found that for adults aged 50 and older or those with underlying health conditions, NMV-r administered within five days of a mild-to-moderate COVID-19 diagnosis provides similar protection against hospitalization as three or more doses of the monovalent mRNA vaccine alone. However, the greatest protection was achieved with both NMV-r treatment and vaccination [24]. The second study, a meta-analysis of seven observational studies with 224,238 vaccinated patients, supports these findings. NMV-r reduced the risk of hospitalization or death in vaccinated patients with mild-to-moderate COVID-19 compared to those who did not receive NMV-r, with greater effectiveness observed in patients aged 50–65 than in those over 65. Despite the protective effect of vaccines, NMV-r still offered significant clinical benefits. Concerns about a viral rebound phenomenon were noted, but evidence suggests this occurs infrequently and with mild symptoms [25].

### 4.2. Hepatitis

Ritonavir, when used as a booster in combination with other direct-acting antivirals, has shown high effectiveness and safety in treating hepatitis C. A real-world study involving 58 patients with chronic hepatitis or compensated hepatic cirrhosis and genotype 1 HCV infection treated with ritonavir-boosted paritaprevir and ombitasvir for 12 weeks reported a sustained virological response at 24 weeks of 96.6%, with no severe adverse events and only mild-to-moderate adverse events [26]. The combination of ombitasvir/paritaprevir/ritonavir plus dasabuvir, with or without ribavirin, has proven effective in treating HCV genotype 1 infection [27]. Clinical trials, such as the AVIATOR study, provided critical data for optimizing dosages and combinations, resulting in high SVR rates and low relapse rates [28]. Overall, ritonavir-boosted therapies offer a robust option for managing hepatitis C, particularly in patients with genotype 1 and 4 infections, and even in those with complex conditions like renal impairment [29] and post-transplant scenarios [30]. The combination regimens demonstrate high efficacy and manageable safety profiles, both in clinical trials and real-world settings.

Chronic hepatitis E virus (HEV) infection predominantly occurs in immunocompromised patients, such as organ transplant recipients, HIV-infected patients, those receiving anticancer therapy, and those on immunosuppressants [31]. Ritonavir was shown on screening tests to have the potential to inhibit HEV growth [32]. A recent study has found that ritonavir inhibits HEV internalization, a crucial point in the life cycle of this virus. Ritonavir with ribavirin, which inhibits HEV RNA replication, resulted in potent inhibition of HEV growth in both genotypes HEV-3 and HEV-4 in cultured cells, outperforming ribavirin monotherapy. This combination showed additive effects without significant cytotoxicity and decreased HEV RNA levels to undetectable amounts in both culture supernatants and intracellularly [33]. The study concluded that combining ritonavir and ribavirin offers a novel, effective strategy for treating chronic HEV infection, warranting further in vivo studies.

### 4.3. Toxoplasmosis

Toxoplasmic encephalitis is a severe issue for immunocompromised individuals, especially those with HIV/AIDS, which has a high mortality risk [34]. Lopinavir boosted with ritonavir (L/r) alone and loaded into poly(lactic-co-glycolic acid) (PLGA) nanoparticles was studied in mice infected with the virulent RH strain of *T. gondii*. Treatment with these formulations significantly reduced mortality rates and parasite numbers in the peritoneal fluid and liver. This effectiveness is likely due to the inhibition of crucial aspartyl protease enzymes in *T. gondii*, preventing parasite egress from host cells and thereby reducing infection of new cells. The study also found that L/r treatments caused significant morphological changes, suggesting apoptosis and autophagy, which further reduced the parasite’s ability to invade and reproduce [35]. Other pathogenic diseases where ritonavir can be seen boosting the effect of drugs are lymphatic filariasis and onchocerciasis [36], leishmaniasis [37], and *Histoplasma capsulatum* [38].

Table 1 summarizes the infectious diseases where ritonavir has been shown to be a potential drug for repurposing, along with the effects and modes of action.

## 5. Cancer Therapy: Mechanisms and Studies Exploring Ritonavir’s Role

### 5.1. Prostate Cancer

Despite the limited success of drugs targeting multidrug resistance in clinical settings, prior research has shown that ritonavir can enhance the bioavailability of oral docetaxel. This study aimed to determine whether combining ritonavir with docetaxel could increase antitumor effects in docetaxel-resistant prostate cancer cells. The single-agent activity of docetaxel, ritonavir, and their combination was tested in 15 tumor cell lines. No synergy was seen in prostate cancer cell lines 22Rv1, DU-145, PC-3, and PC-3M [39]. This contrasted with previous findings that suggested a synergistic effect between docetaxel and ritonavir in DU-145 cells at low concentrations. This investigation pointed to the fact that ritonavir reduced the robust nuclear factor kappa B (NFκB) DNA binding activity of DU-145 cells both in vitro and in vivo as a potential mechanism of cytotoxicity of ritonavir in these cells [40]. Prostate cancer cells, among other cancers, frequently exhibit hyperactivity of the NFκB pathway, which may confer a degree of resistance to chemotherapy on these malignant cells [41]. Additionally, in the DU-145 proliferation assay, ritonavir alone showed cytotoxic activity at an IC_50_ concentration of 3 µM, which other groups did not confirm.

Resistant prostate cancer cell lines DU-145DOC10 and 22Rv1DOC8 were established through prolonged exposure to docetaxel. Genome-wide RNA sequencing revealed significant upregulation of adenosine triphosphate-binding cassette, subfamily B, member 1 (ABCB1) in these resistant sublines. This is the gene that encodes P-gp and is usually overexpressed in cancers, causing the development of resistance to anticancer drugs due to increased excretion of them from the cells due to increased P-gp transporters [42], and there was a confirmed increase in P-gp expression. The addition of 10 µM ritonavir reversed docetaxel resistance in DU-145DOC10, while 32 µM ritonavir was required to achieve similar effects in 22Rv1DOC8. Inhibition of P-gp with elacridar also reversed docetaxel sensitivity, confirming the role of P-gp in mediating resistance [39]. Other P-gp inhibitors have also been demonstrated to reverse taxane resistance in prostate cancer. One example is itraconazole, an antifungal, which reverses docetaxel resistance in various prostate cell lines using concentrations of around 2.5–5 µM, slightly lower than ritonavir [43].

The study also explored cabazitaxel, which is supposed to have a lower affinity for P-gp [44]. Although preclinical studies suggested cabazitaxel’s potency in P-gp-associated resistant cell lines, the study confirmed that cabazitaxel is indeed a P-gp substrate. Increased P-gp expression was identified as a driver of cabazitaxel resistance, and knocking down ABCB1 restored sensitivity. Combining cabazitaxel with ritonavir showed the same synergistic effects in docetaxel-resistant cell lines as seen with docetaxel. Overall, the study demonstrates that ritonavir reverses resistance to both docetaxel and cabazitaxel in prostate cancer cell lines by inhibiting P-gp-mediated drug efflux [39].

The use of ritonavir as a booster for taxane anticancer drugs has been explored in clinical trials. Several doses of new solid formulations of docetaxel ModraDoc001 (10 mg docetaxel, freeze-dried) and ModraDoc006 (10 mg docetaxel, spray-dried) with ritonavir (100 mg) were tested first in a phase I clinical trial in patients with diverse cancer types (NCT01173913). The maximum tolerated dose (MTD) was determined to be 20/20 mg twice weekly for ModraDoc001 capsules and 30/20 mg twice weekly for ModraDoc006 tablets. Treatment-related toxicity was mainly grade 1 or 2 and manageable with dose adjustments. No unexpected safety issues were noted, consistent with the known safety profile of intravenous (IV) docetaxel. The docetaxel exposure at the MTD for the ModraDoc006 tablet formulation was comparable to once-weekly IV docetaxel. The interpatient variability for ModraDoc006 was like that of IV docetaxel. Promising antitumor activity was observed. Because of this, ModraDoc006 and ritonavir were chosen for further exploration [45]. This occurred in a phase II study of this drug boosted with ritonavir in metastatic castration-resistant prostate cancer (mCRPC) patients (NCT04028388). ModraDoc006/r demonstrated a favorable safety profile and comparable efficacy to IV docetaxel in mCRPC patients, supporting the further development and expansion of clinical trials comparing ModraDoc006/r to the best available therapies for refractory mCRPC [46].

### 5.2. Ovarian Cancer

Ovarian cancer, a deadly gynecologic malignancy with poor five-year survival rates, is a focus of intense research for new antineoplastic compounds, particularly for drug-resistant and relapsing cases where current chemotherapy options are often ineffective [47]. A study showed for the first time that ritonavir acts as an effective anti-proliferative agent for ovarian cancer cell lines MDAH- 2774 and SKOV-3 [48].

The retinoblastoma protein (RB) is a crucial tumor suppressor controlling progression through the G1 phase of the cell cycle [49]. Elevated levels of under-phosphorylated RB in ritonavir-treated cells suggest lower levels of CDK-2, 4, and 6—the proteins responsible for cell cycle progression via RB phosphorylation [50]. Gene profile analysis confirmed the downregulation of CDKs and cyclins, gatekeepers of the G0/G1 phase. Ritonavir treatment also resulted in lower levels of CDK inhibitors and RB phosphorylation, along with increased expression of the E2F-1 transcription factor, corroborating reduced S-phase entry by over 25% [48]. These results were also observed in an in vitro study with A2780 ovarian cancer cells and in vivo mice models [51].

The PI3K/AKT pathway is overexpressed in ovarian and other common cancers, contributing to chemotherapy resistance, and inhibition of AKT sensitizes chemoresistant cells to cisplatin-induced apoptosis [52]. This study corroborates that ritonavir inhibits AKT phosphorylation, promoting effective apoptosis. These findings suggest ritonavir’s potential as an anti-AKT agent, particularly for relapsed ovarian cancer patients with drug-resistant phenotypes. Bcl-2 inhibition, mediated through an AKT-dependent pathway, showed similar results with anti-AKT siRNA treatment [48].

A major reason for high ovarian cancer mortality is the late-stage diagnosis (FIGO III), where tumor cells have often spread through the peritoneal cavity [53]. Ritonavir’s inhibition of invasion and migration in ovarian cancer cell lines adds to its anticancer properties and may be especially useful against trans-peritoneal spread. The results above were obtained at concentrations between 5 and 20 μM, which are achievable blood plasma concentrations of ritonavir in patients that typically reach 15 μM, with higher concentrations observed in some individuals [48].

### 5.3. Lung Cancer

Ritonavir has demonstrated the ability to inhibit the growth of the NCI-H522 human lung adenocarcinoma cell line with an IC_50_ of 42 ± 2 µM. Ritonavir induces a G0/G1 cell cycle arrest and promotes apoptosis in a time-dependent manner. G1 arrest caused by ritonavir in the H522 cell line is partially mediated by the downregulation of heat shock protein 90 (Hsp90) and the consequent reduction in CDK4 [54]. Hsp90 is often overexpressed in lung cancer and is involved in the progression of the cell cycle by the activation of a cascade of molecules, which includes CDK4 activation for G1/S passage [55]. Similar reductions in Hsp90 and CDK4 were observed in the A549 and H460 non-small-cell lung cancer (NSCLC) lines at 7.8 µM; below the median clinical serum concentration (Cmax) of 27 µM, it is suggested that clinical doses of ritonavir could potentially inhibit Hsp90 function. Additionally, ritonavir sensitizes H522, A549, and H460 cell lines to the S-phase active chemotherapeutic agent gemcitabine. These findings propose Hsp90 and CDK4 as novel targets of ritonavir in NSCLC and suggest that ritonavir’s inhibition of CDK4 may increase the sensitivity of NSCLC cells to DNA replication-associated stress [54].

Another study also investigated the potential of ritonavir as a therapeutic agent against lung adenocarcinoma. The research focused on identifying the mechanisms and molecular targets of ritonavir in lung cancer cells. Ritonavir was found to inhibit the growth of both K-ras mutant and wild-type lung adenocarcinoma cell lines, specifically using the A549, H460, and H522 cell models. The drug induced cell cycle arrest at the G0/G1 phase and promoted apoptosis. This was accompanied by a significant downregulation of CDKs, cyclin D1, and phosphorylated retinoblastoma protein (pRb). Additionally, ritonavir reduced the levels of survivin mRNA and protein by more than two-fold. All of this was achieved with concentrations between 20 and 40 µM [56]. Survivin is a protein involved in inhibition of apoptosis and regulation of cell division, making it a crucial target for ritonavir’s anticancer activity [57]. This decrease was achieved by the inhibition of the phosphorylation of c-Src and signal transducer and activator of transcription 3 (STAT3), both of which are important for survivin gene expression and cell growth [58].

Combination studies with chemotherapy agents showed that ritonavir exhibited synergistic effects with gemcitabine and additive effects with cisplatin. The triple combination of ritonavir, gemcitabine, and cisplatin was synergistic in the A549 cell line and additive in the H522 cell line, suggesting potential clinical benefits at feasible ritonavir concentrations (15–20 µM). The findings highlight the potential of ritonavir as a therapeutic agent for lung adenocarcinoma, with survivin identified as an important target and potential biomarker for ritonavir sensitivity. The drug’s combination with gemcitabine and cisplatin might enhance treatment efficacy and could be explored further in clinical trials [56].

### 5.4. Breast Cancer

With a median survival from 9 months to 3 years, conventional therapies for recurrent or metastatic breast cancer are not very effective [59]. Therefore, a study looked at the possibility of ritonavir as a therapeutic agent [60]. An increase in AKT, often associated with the PI3K/AKT/mTOR signaling pathway, is essential for the survival of several breast cancer cell lines due to its increase in protein synthesis, cell growth, and survival. The discovery of new inhibitors of this molecule is an interesting path of research [61]. Breast cancer cells, including MDA-MB-231, MCF7, and T47D, were examined in this work to determine how well ritonavir can reduce breast cancer cell growth and cause apoptosis [60].

Among these breast cancer lines, ritonavir exhibits varying degrees of sensitivity. In particular, estrogen receptor (ER)-positive cells (T47D and MCF7) have lower IC_50_ values than ER-negative lines (12–24 µmol/L vs. 45 µmol/L), possibly due to a depletion of the ER-α [60]. This receptor is often associated with epithelial-to-mesenchymal transition and drug resistance in breast cancer, and its inhibition leads to cell apoptosis [62].

In S + G2-M phase cells, ritonavir caused G1 arrest, lowered AKT phosphorylation, and lowered clonogenic efficiency. Furthermore, by binding to Hsp90, ritonavir partly inhibited its chaperone activity and caused the degradation of various Hsp90-associated proteins, including CDKs. These results imply that ritonavir’s suppression of the growth of breast cancer cells might be facilitated by its influence on the AKT pathway and Hsp90 activity. Ritonavir also slowed tumor development at therapeutically feasible serum levels and decreased intratumoral AKT activation, according to in vivo studies using MDA-MB-231 xenografts. Moreover, ritonavir’s anti-proliferative effects are amplified when combined with Hsp90 RNA interference, suggesting that ritonavir’s efficacy in treating breast cancer may be enhanced by targeting Hsp90. The results are in favor of ritonavir’s possible clinical development as a new treatment drug for breast cancer, particularly when used in conjunction with Hsp90 pathway-targeting tactics [60].

Ritonavir is of particular interest in breast cancer due to its docetaxel-enhancing capabilities. In a first study, CYP3A knockout mice were implanted with syngeneic breast tumors that naturally expressed CYP3A. Four groups of mice were created: one for the control group, one for the ritonavir alone, one for the docetaxel alone, and one for the combination of the two drugs. The doses were 20 mg/kg docetaxel once a week and 12.5 mg/kg ritonavir orally for five subsequent days per week. In comparison to docetaxel therapy alone, the co-administration of ritonavir with docetaxel resulted in a 2.5-fold larger area under the curve (AUC) of docetaxel. This increase in the concentration of docetaxel in tumor tissues was shown to be significant. As seen from the smaller tumors and longer life in the co-treated group, this rise in intratumoral docetaxel concentration increased the antitumor activity. Additionally, ritonavir alone was shown to have a slight, yet substantial, antitumor impact by the study [63].

The researchers also created a tumor growth inhibition model that included the effects of both drugs and a PK model to characterize the systemic and tumor-specific concentrations of docetaxel and ritonavir. The greater antitumor impact was primarily caused by the higher concentration of docetaxel in the tumor, and it was discovered that ritonavir reduced the systemic clearance of docetaxel by 8% in the co-treated group [63]. This could be related to the ability of ritonavir to inhibit other drug transporters or metabolizing enzymes that might affect the systemic exposure of docetaxel [64]. The conclusion that the combination of docetaxel and ritonavir offers a synergistic effect in inhibiting tumor growth is supported by the PK-PD model, which accurately described the observed tumor volume profiles. Ritonavir contributes to this effect by both increasing the concentration of docetaxel in the tumor and directly preventing tumor growth [63].

In a related investigation, the same research team looked at how the same combination affected immunocompetent mouse models of breast cancer. The purpose of this study was to determine whether ritonavir may increase the anticancer activity of docetaxel when given intravenously by reducing its metabolism within tumors, and the dosages were 20 mg/ kg IV docetaxel and 12.5 mg/kg oral ritonavir. The findings were consistent with the previous trial, in that the docetaxel and ritonavir combination had a noticeable impact. More specifically, compared to therapy with docetaxel alone, this combination reduced tumor size and considerably increased the mice’s median survival time from 54 days to 66 days. Furthermore, it was discovered that ritonavir decreased the amount of docetaxel-metabolism-related products in the tumors, indicating that it blocks docetaxel metabolism inside the tumor itself without changing the tumor’s general histology. This study further cements the findings that ritonavir increases the effectiveness of docetaxel by preventing its metabolism within the tumor, which increases the drug’s antitumor action even if it does not directly target the tumor itself [65].

Incorporating ritonavir into delivery systems alongside anticancer drugs can be an effective strategy for combating cancer. For example, one study concentrated on creating stimuli-sensitive, hyaluronic acid (HA)-targeted nanomicelles that co-encapsulate ritonavir and paclitaxel to overcome multidrug resistance in triple-negative breast cancer (TNBC) and metastatic breast cancer (MBC) [66]. Using HA to target CD44 receptors [67], which are overexpressed on the surface of many cancer cells, including those in MBC and TNBC [68], the nanomicelles are engineered to deliver these chemotherapeutic drugs exclusively to cancer cells while reducing their impact on healthy cells.

Although paclitaxel is effective, P-gp-mediated resistance in cancer cells can result in serious adverse effects. As previously mentioned, ritonavir can inhibit P-gp [15], which raises the amount of paclitaxel within cancer cells and aids in the fight against MDR. According to the study, PTX and RTV are efficiently delivered by these nanomicelles to MBC and TNBC cells, increasing the rate of cancer cell apoptosis and decreasing the toxicity to healthy cells. This focused strategy may significantly increase the effectiveness of chemotherapy for aggressive breast tumors that are unresponsive to current therapies [66].

Other drugs have also been used as a booster for anticancer drugs in breast cancer, such as quinidine, which boosted the docetaxel effect at a concentration of around 4 µM. However, it is difficult to make a comparison with ritonavir due to experiments being conducted in vitro vs. in vivo and using drug transporters [69].

### 5.5. Bladder Cancer

Studies showed that the inhibition of Hsp90 leads to the abnormal protein subset of the cancer cell being destabilized, increasing the number of unfolded proteins inside the cell. Endoplasmic reticulum (ER) stress results from this, and persistent or untreated ER stress induces apoptosis [70]. Hence, Hsp90 inhibition presents a compelling new cancer-fighting tactic. One constraining element, though, is that the unfolded proteins that have not been fixed may be broken down by the proteasome and will not build up inside the cell. Thus, to generate enough ER stress to trigger apoptosis, both Hsp90 and the proteasome would need to be suppressed [71]. Ritonavir inhibits Hsp90’s chaperone function by binding to it and also inhibits the proteasome, an effect linked to the buildup of p21, a cyclin-dependent kinase inhibitor [72]. This ER stress caused by ritonavir is interesting to explore for bladder cancer treatment.

A study of the effects of ixazomib and ritonavir on bladder cancer cells focused on the mechanisms of ER stress and ubiquitinated protein accumulation explained above [73]. The study hypothesized that ixazomib, a proteasome inhibitor [74], in conjunction with ritonavir may kill bladder cancer cells by stopping the breakdown of ubiquitinated proteins, which would cause ER stress and death. The results show that at doses of 40 µM ritonavir and 100 µM ixazomib, the combination of the two drugs strongly promotes apoptosis, disrupts the cell cycle, and lowers cell viability in bladder cancer cell lines UMUC3, J82, and 5637. Combination treatment reduced cyclin D1 and CKD4 expression while raising the sub-G1 percentage. In support of increased apoptosis, the therapy also raises the levels of proteins linked to apoptosis, including cleaved PARP, activated caspase 3, and NOXA. Additional investigation reveals that the combination of ritonavir and ixazomib significantly increases ubiquitinated protein accumulation and ER stress, which is corroborated by elevated expression of ER stress indicators, such as glucose-regulated protein 78 (GRP78) and Hsp70 [73,75]. According to the study, the accumulated ubiquitinated proteins and the consequent ER stress play a crucial role in the anticancer effects that have been seen. According to the study’s findings, ritonavir with ixazomib may be used to create innovative treatments for bladder cancer that target the buildup of ubiquitinated proteins [73].

The same group later conducted similar experiments but used ritonavir with nelfinavir, which is another HIV protease inhibitor that can induce ER stress. The same bladder cancer cells were treated with varying concentrations of nelfinavir and ritonavir. The results demonstrated that the combination significantly inhibited cell growth, reduced colony formation, and induced apoptosis more effectively than either drug alone. The combination therapy induced ER stress synergistically, as evidenced by elevated levels of GRP78, ER-resident protein 44 (ERP44), and endoplasmic oxidoreductin-1-like protein (Ero1L). Furthermore, it inhibited the mTOR pathway by increasing AMP-activated protein kinase (AMPK) expression and dephosphorylating S6 ribosomal protein. Histone acetylation was also enhanced due to decreased expression of histone deacetylases (HDACs) 1, 3, and 6. The study concluded that the combination of nelfinavir and ritonavir effectively kills bladder cancer cells through multiple mechanisms, including ER stress induction, mTOR pathway inhibition, and histone acetylation, making it a promising therapeutic strategy for bladder cancer [76]. Further studies from this group included the combination of ritonavir with lopinavir [77] and a dual histone deacetylase-proteasome inhibitor RTS-V5 [78], with a demonstrated increase in ER stress and consequent decrease in cell viability in bladder cancer, as well as renal cancer cells for lopinavir and ritonavir combination.

### 5.6. Pancreatic Cancer

Because of ritonavir’s previously documented ability to slow down cell growth and trigger apoptosis in ovarian cancer, the authors investigated the drug’s potential as an anticancer agent, with a focus on pancreatic ductal adenocarcinoma (PDAC). Ritonavir was demonstrated to reduce the development of PDAC cell lines BxPC-3, MIA PaCa-2, and PANC-1 in a concentration-dependent manner (5–20 µM), predominantly inducing cell death via the intrinsic apoptotic mechanism. Additionally, the study discovered that ritonavir increases the expression of genes that block the cell cycle and downregulates the genes that promote it, especially those that improve the connection between the transcription factor E2F-1 and the tumor suppressor RB [79]. Through this connection, the phosphorylation of RB is inhibited, which sequesters E2F-1 and downregulates the genes required for the cell cycle’s S-phase development, ultimately preventing cell proliferation [80].

Furthermore, ritonavir was demonstrated to block the AKT pathway, which adds to its antitumor effects, much like it did for the other cancers analyzed in this review. Ritonavir and gemcitabine, a common chemotherapy drug for pancreatic cancer, when taken together led to a significant increase in cell death as compared to when taken separately, indicating a synergistic impact of ritonavir and gemcitabine. The study’s findings suggest that ritonavir may be used in combination therapy to treat pancreatic cancer [79].

Verapamil is a calcium channel blocker with P-gp inhibition properties and has also been tested for repurposing for pancreatic cancer treatment as a gemcitabine booster. Combination synergy in decreased cell viability and increased apoptosis were obtained but with concentrations of and above 50 µM of verapamil, above the 5–20 µM values obtained with ritonavir acting alone. This shows the superiority of ritonavir for pancreatic cancer repurposing [81].

In addition to treatment, ritonavir has been explored in pancreatic cancer as an imaging agent. Cathepsin E (CTSE) is a molecule overexpressed in invasive PDAC and has emerged as a target for imaging due to its specificity and the fact that it only exists intracellularly [82]. Ritonavir has an affinity for this molecule, and therefore, a study was conducted to develop and test a small molecule based on a chemical modification of ritonavir to use as an imaging agent during surgical removal of this type of pancreatic tumor. The results showed that the compound, called RIT-TMB, had an affinity for CTSE at low concentrations (around 40 nM) and that it co-localized correctly with tumors in in vivo models. This allows the imaging of individual cancer cells and helps in surgery, as well as in diagnostic and other procedures [83].

### 5.7. Multiple Myeloma

More and more people are realizing how important metabolic adaptability is in the development of tumors, especially the connection between glucose metabolism and cellular viability. To power the anabolic pathways required for cell proliferation and to become resistant to chemotherapy, tumor cells use more glucose [84]. According to a study, glucose transporter type 4 (GLUT4), which is constitutively active on the cell surface, is crucial for multiple myeloma (MM) cell proliferation. Moreover, researchers discovered a connection between GLUT4 activity and the preservation of Mcl-1, a protein essential for the survival of MM cells. Reduction in Mcl-1 expression, which is required for cell death but insufficient on its own, is achieved by inhibiting GLUT4 [85]. Protease degradation and mTOR-dependent translation are not involved in the transcriptional control of Mcl-1 by GLUT4. Given that Mcl-1 is linked to poor prognosis in MM, this suggests a unique treatment approach [86].

Ritonavir showed off-target effects on metabolism, particularly in GLUT4 inhibition, which can reduce MM cell proliferation and increase chemosensitivity. Although the metabolic side effects of ritonavir are concerning, they may be leveraged for MM treatment, perhaps with variable dosage regimens to reduce negative effects [85].

Ritonavir-treated MM cells can become resistant to the drug despite its selectivity by becoming more dependent on mitochondrial oxidative phosphorylation, especially through compensatory glutamine metabolism. Metformin, a mitochondrial complex I inhibitor that has shown anticancer activity in both in vitro and in vivo investigations, can overcome this resistance. Ritonavir and metformin together efficiently inhibited important survival pathways in MM cells (KMS11, L363, and JJN3), such as the AKT and mTORC1 pathways that control the creation of Mcl-1 [87].

### 5.8. Glioblastoma

The brain tumor known as glioblastoma (GBM) is extremely aggressive and deadly. Even with the usual treatment of surgery, temozolomide (TMZ) chemotherapy, and radiation therapy, glioblastoma is a notoriously difficult cancer to treat and has poor prognosis [88].

In the first research for ritonavir repurposing in GBM, the investigators examined multiple drugs: antiviral drugs, such as ritonavir (25 µM), as well as aprepitant, an anti-nausea drug. Their objective was to ascertain whether the co-administration of these drugs with TMZ may heighten the cytotoxic impact of TMZ on glioma cells. According to the findings, each of the three drugs—TMZ, ritonavir, and aprepitant—exhibited a slight growth inhibition. Interestingly, there was an additional growth inhibition observed when TMZ was combined with ritonavir or aprepitant. The most noteworthy discovery was the synergistic action of ritonavir with aprepitant, which led to a 64% suppression of glioma cell proliferation. The growth inhibition increased to 78% when all three drugs were used together [89].

In another study, the researchers conducted both in vitro and in vivo tests to investigate the effects of ritonavir alone and in combination with TMZ and radiation. They used patient-derived glioblastoma cells (pGBMs) and created GBM cell lines to evaluate cell survival, migration, invasion, and indicators of autophagy and ER stress. The effects of ritonavir in conjunction with TMZ or radiation on tumor development and overall survival were investigated using mouse xenograft models. At clinically relevant doses (21–23 µM), ritonavir was reported to demonstrate selective cytotoxic effects on GBM cells, resulting in a substantial reduction in cell viability in pGBMs and established cell lines while sparing non-cancerous cells. In GBM cells, ritonavir therapy elevated the indicators of ER stress and autophagy. When paired with TMZ or radiation, ritonavir increased the antitumor effects, leading to larger cell viability and migratory decreases and a longer lifespan in the mouse models. Additionally, ritonavir impacted the tumor microenvironment, perhaps by modifying immune responses and decreasing angiogenesis, which further contributed to its antitumor effects [90].

The combination of lopinavir and ritonavir, usually used to impede the transmission of HIV from mother to child, was also tested for GBM in the U-87 MG cell line. The findings demonstrated that co-treatment at higher concentrations (25 and 50 µM) of lopinavir and ritonavir resulted in a significant increase in ROS generation, damaged the mitochondrial network, and caspase-independently promoted apoptosis, although this was achieved at above clinically relevant concentrations [91]. Another obstacle is the low distribution of ritonavir to cerebrospinal fluid, at about 0.05 mg/L, although this test to evaluate central nervous system levels of ritonavir may not be the best, since it does not account for the low levels of proteins present; therefore, this needs more study [92].

Table 2 shows a summary of all the cancers ritonavir was tested in for repurposing, along with the main effects and demonstrated pathways.

## 6. Conclusions

The goal of this review was to explore the potential repurposing of ritonavir, a well-known HIV protease inhibitor, for the treatment of cancer and other infectious diseases. Ritonavir was first created to treat HIV/AIDS, but it has shown a lot of promise outside of its initial use. This is mainly because of its capacity to inhibit P-glycoprotein and cytochrome P450 3A4, which increases the efficacy of other treatment drugs.

The information gathered here highlights the potential of ritonavir in cancer therapy, as it has demonstrated success in treating a variety of cancer type models, including ovarian, lung, breast, and bladder tumors, as well as in overcoming drug resistance. Research on ritonavir in oncology is justified because of the ways by which it exhibits anticancer effects, including apoptosis induction, cell cycle arrest, and treatment sensitization of cancer cells.

In addition to its oncologic potential, this review also looked at the use of ritonavir in the treatment of various infectious diseases, such as hepatitis virus infection and the COVID-19 pandemic. Ritonavir’s effective inclusion in the COVID-19 Paxlovid treatment regimen is an excellent example of how repurposed drugs may be used to meet critical public health requirements.

Overall, this review highlights the significance of repurposing existing drugs, such as ritonavir, to address novel treatment difficulties. According to the results, ritonavir may prove to be an effective treatment for illnesses and resistant tumors. However, most of these works used in vitro models, and the results need to be validated with preclinical, and afterward—and if promising results are obtained—clinical studies to thoroughly explore this promising treatment option.

## Figures and Tables

**Table 1:** Repurposing opportunities of ritonavir in infectious diseases.

**Disease**	**Model**	**Main findings**	**Ref**
COVID-19	Clinical trials and observational studies in high-risk, unvaccinated, and vaccinated COVID-19 outpatients	- Reduced hospitalization and death in high-risk patients when combined with nirmatrelvir (Paxlovid). - Effective when administered within five days of symptom onset. - Beneficial for vaccinated patients, especially those between 50 and 65.	[19,22,23]
Hepatitis C	Patients with chronic hepatitis or compensated hepatic cirrhosis and genotype 1 HCV infection	- Ritonavir-boosted paritaprevir and ombitasvir with 96.6% sustained virological response at 24 weeks in HCV genotype 1 infection. - Effective, with manageable safety profiles.	[24,25,26]
Hepatitis E	Cell culture studies with HEV-3 and HEV-4 genotypes	- Inhibited HEV growth and internalization when combined with ribavirin. - Reduced HEV RNA levels to undetectable amounts without significant cytotoxicity.	[30,31]
Toxoplasmosis	Mouse model infected with virulent RH strain of *T. gondii*	- Lopinavir boosted with ritonavir (L/r), both alone and in PLGA nanoparticles, significantly reduced mortality. - Reduced parasite numbers and induced morphological changes leading to apoptosis and autophagy in the parasite.	[33]

**Table 2:** Repurposing opportunities of ritonavir in various cancer types.

**Cancer Type**	**Model**	**Main Findings**	**Ref**
Prostate Cancer	Normal (22Rv1, DU-145, PC-3, PC-3M) and resistant (DU-145DOC10, 22Rv1DOC8) prostate cancer cell lines	- No synergy in prostate cells non-resistant to docetaxel. - Reversal of resistance to docetaxel and cabazitaxel via P-gp inhibition.	[37]
	Phase I cancer patients and phase II mCRPC patients	- New formulation of docetaxel/ritonavir for oral administration (ModraDoc006).	[42,43]
Ovarian Cancer	Ovarian cancer cell lines (e.g., MDAH- 2774, SKOV-3, A2780)	- Elevated levels of under-phosphorylated RB in ritonavir-treated cells indicate reduced CDK-2, 4, and 6 activities, leading to cell cycle arrest in the G1 phase. - Inhibition of AKT phosphorylation, sensitizing chemoresistant cells to cisplatin-induced apoptosis and inhibiting Bcl-2. - Reduction in migration.	[45,48]
Lung Cancer	NSCLC cell lines (H522, A549, H460)	- G1 arrest via reduction in Hsp90 levels, leading to decreased CDK4 activity. - Increased sensibilization to gemcitabine and synergism with gemcitabine and cisplatin. - Downregulates CDKs, cyclin D1, phosphorylated retinoblastoma protein (pRb), and survivin mRNA/protein. - Inhibiting phosphorylation of c-Src and STAT3, reducing survivin expression, crucial for inhibiting apoptosis and regulating cell division.	[51,53]
Breast Cancer	Breast cancer cell lines (MDA-MB-231, MCF7, and T47D)	- Growth inhibition and apoptosis, especially in ER-positive cells. - G1 cell cycle arrest. - Lowers Akt phosphorylation and reduces Hsp90 activity, leading to decreased CDK levels. - Reduces survivin mRNA/protein levels and decreases intratumoral Akt activation.	[57]
	Mouse models with implanted breast cancer tumors	- Enhanced docetaxel’s antitumor activity by increasing intratumoral concentration. - Co-administration leads to smaller tumors and longer survival in mouse models. - Reduced systemic clearance of docetaxel and blocked its metabolism within tumors.	[60,62]
	MBC and TNBC cancer cell lines	- Co-encapsulates ritonavir and paclitaxel in HA-targeted nanomicelles. - Targets CD44 receptors on cancer cells, delivering drugs efficiently and reducing impact on healthy cells. - Increases cancer cell apoptosis and overcomes P-gp-mediated drug resistance.	[63]
Bladder Cancer	Bladder cancer cell lines (UMUC3, J82, 5637)	- Inhibits both Hsp90 and the proteasome, leading to ER stress and accumulation of p21, promoting apoptosis. - Increased apoptosis-related proteins (cleaved PARP, caspase 3, NOXA) and ER stress indicators (GRP78, Hsp70). - Combinations with ixazomib or nelfinavir or lopinavir or RTS-V5 all decreased cell viability and caused ER stress.	[68,69,72,73,74]
Pancreatic Cancer	Pancreatic ductal adenocarcinoma cell lines (BxPC-3, MIA PaCa-2, PANC-1)	-. Inhibits phosphorylation of RB, sequestering E2F-1 and preventing S-phase progression, reducing cell proliferation. - Blocks the AKT pathway, enhancing antitumor effects. - Synergy with gemcitabine.	[75]
	In vivo tumor models	- Modified ritonavir compound (RIT-TMB) developed for imaging PDAC during surgery. - Targets cathepsin E and enables precise imaging of cancer cells in in vivo tumor models.	[78]
Multiple Myeloma	Multiple myeloma cell lines (KMS11, L363, JJN3)	- Inhibition of GLUT4, reducing Mcl-1 expression, leading to cell death and increased chemosensitivity. - Combination with metformin decreases ritonavir resistance and inhibits AKT and mTORC1, key for cancer survival.	[80,82]
Glioblastoma	Glioblastoma cell lines (e.g., U-87 MG, primary glioma cells) and mouse model	- In combination with TMZ, aprepitant, or radiation, significantly reduces cell viability and proliferation. - Induces ER stress and autophagy. - Combination with lopinavir enhances ROS generation and apoptosis in glioma cells.	[84,85,86]

## Data Availability

Not applicable.

## References

[1] DrugBank Ritonavir. https://go.drugbank.com/drugs/DB00503.

[2] Schmit J.C., Ruiz L., Clotet B., Raventos A., Tor J., Leonard J., Desmyter J., De Clercq E., Vandamme A.M. (1996). Resistance-related mutations in the HIV-1 protease gene of patients treated for 1 year with the protease inhibitor ritonavir (ABT-538). Aids.

[3] Kempf D.J., Norbeck D.W., Codacovi L., Wang X.C., Kohlbrenner W.E., Wideburg N.E., Paul D.A., Knigge M.F., Vasavanonda S., Craig-Kennard A. (1990). Structure-based, C2 symmetric inhibitors of HIV protease. J. Med. Chem..

[4] Whitesell J.K. (1989). C2 symmetry and asymmetric induction. Chem. Rev..

[5] Hull M.W., Montaner J.S. (2011). Ritonavir-boosted protease inhibitors in HIV therapy. Ann. Med..

[6] FDA NORVIR (Ritonavir). www.accessdata.fda.gov/drugsatfda_docs/label/2017/209512lbl.pdf.

[7] Lledó-García R., Nácher A., Prats-García L., Casabó V.G., Merino-Sanjuán M. (2007). Bioavailability and pharmacokinetic model for ritonavir in the rat. J. Pharm. Sci..

[8] Danner S.A., Carr A., Leonard J.M., Lehman L.M., Gudiol F., Gonzales J., Raventos A., Rubio R., Bouza E., Pintado V. (1995). A short-term study of the safety, pharmacokinetics, and efficacy of ritonavir, an inhibitor of HIV-1 protease. European-Australian Collaborative Ritonavir Study Group. N. Engl. J. Med..

[9] Larson K.B., Wang K., Delille C., Otofokun I., Acosta E.P. (2014). Pharmacokinetic enhancers in HIV therapeutics. Clin. Pharmacokinet..

[10] EMA Norvir. www.ema.europa.eu/en/documents/product-information/norvir-epar-product-information_en.pdf.

[11] Greenblatt D.J., Harmatz J.S. (2015). Ritonavir is the best alternative to ketoconazole as an index inhibitor of cytochrome P450-3A in drug–drug interaction studies. Br. J. Clin. Pharmacol..

[12] Loos N.H.C., Beijnen J.H., Schinkel A.H. (2022). The Mechanism-Based Inactivation of CYP3A4 by Ritonavir: What Mechanism?. Int. J. Mol. Sci..

[13] Marzolini C., Gibbons S., Khoo S., Back D. (2016). Cobicistat versus ritonavir boosting and differences in the drug–drug interaction profiles with co-medications. J. Antimicrob. Chemother..

[14] Loos N.H.C., Beijnen J.H., Schinkel A.H. (2023). The inhibitory and inducing effects of ritonavir on hepatic and intestinal CYP3A and other drug-handling proteins. Biomed. Pharmacother..

[15] Drewe J., Gutmann H., Fricker G., Török M., Beglinger C., Huwyler J. (1999). HIV protease inhibitor ritonavir: A more potent inhibitor of P-glycoprotein than the cyclosporine analog SDZ PSC 833. Biochem. Pharmacol..

[16] Storch C.H., Theile D., Lindenmaier H., Haefeli W.E., Weiss J. (2007). Comparison of the inhibitory activity of anti-HIV drugs on P-glycoprotein. Biochem. Pharmacol..

[17] Gupta A., Zhang Y., Unadkat J.D., Mao Q. (2004). HIV protease inhibitors are inhibitors but not substrates of the human breast cancer resistance protein (BCRP/ABCG2). J. Pharmacol. Exp. Ther..

[18] Annaert P., Ye Z.W., Stieger B., Augustijns P. (2010). Interaction of HIV protease inhibitors with OATP1B1, 1B3, and 2B1. Xenobiotica.

[19] Zhang Y., Tang L.V. (2021). Overview of Targets and Potential Drugs of SARS-CoV-2 According to the Viral Replication. J. Proteome Res..

[20] Reis S., Metzendorf M.I., Kuehn R., Popp M., Gagyor I., Kranke P., Meybohm P., Skoetz N., Weibel S. (2023). Nirmatrelvir combined with ritonavir for preventing and treating COVID-19. Cochrane Database Syst. Rev..

[21] Hammond J., Leister-Tebbe H., Gardner A., Abreu P., Bao W., Wisemandle W., Baniecki M., Hendrick V.M., Damle B., Simón-Campos A. (2022). Oral Nirmatrelvir for High-Risk, Nonhospitalized Adults with Covid-19. N. Engl. J. Med..

[22] FDA Fact Sheet for Healthcare Providers: Emergency Use Authorization for Paxlovid. www.fda.gov/media/155050/download.

[23] WHO Therapeutics and COVID-19: Living Guideline, 10 November 2023. https://app.magicapp.org/#/guideline/nBkO1E.

[24] Shah M.M., Joyce B., Plumb I.D., Sahakian S., Feldstein L.R., Barkley E., Paccione M., Deckert J., Sandmann D., Hagen M.B. (2024). Combined Protection of Vaccination and Nirmatrelvir-Ritonavir against Hospitalization in Adults with Coronavirus Disease 2019. Clin. Infect. Dis..

[25] Li H., Xiang H., He B., Zhang Q., Peng W. (2023). Nirmatrelvir plus ritonavir remains effective in vaccinated patients at risk of progression with COVID-19: A systematic review and meta-analysis. J. Glob. Health.

[26] Miyasaka A., Yoshida Y., Yoshida T., Murakami A., Abe K., Ohuchi K., Kawakami T., Watanabe D., Hoshino T., Sawara K. (2018). The Real-world Efficacy and Safety of Ombitasvir/Paritaprevir/Ritonavir for Hepatitis C Genotype 1. Intern. Med..

[27] Flisiak R., Flisiak-Jackiewicz M. (2017). Ombitasvir and paritaprevir boosted with ritonavir and combined with dasabuvir for chronic hepatitis C. Expert. Rev. Gastroenterol. Hepatol..

[28] Poordad F., Agarwal K., Younes Z., Cohen D., Xie W., Podsadecki T. (2015). Low relapse rate leads to high concordance of sustained virologic response (SVR) at 12 weeks with SVR at 24 weeks after treatment with ABT-450/ritonavir, ombitasvir, and dasabuvir plus ribavirin in subjects with chronic hepatitis C virus genotype 1 infection in the AVIATOR study. Clin. Infect. Dis..

[29] Abd-Elsalam S., Abo-Amer Y.E., El-Abgeegy M., Elshweikh S.A., Elsergany H.F., Ahmed R., Elkadeem M., Hawash N., Soliman S., Badawi R. (2020). Efficacy and safety of ombitasvir/paritaprevir/ritonavir/ribavirin in management of Egyptian chronic hepatitis C virus patients with chronic kidney disease: A real-life experience. Medicine.

[30] Tronina O., Durlik M., Wawrzynowicz-Syczewska M., Buivydiene A., Katzarov K., Kupcinskas L., Tolmane I., Karpińska E., Pisula A., Karwowska K.M. (2017). Real-World Safety and Efficacy of Ombitasvir/Paritaprevir/Ritonavir/+Dasabuvir±Ribavirin (OBV/PTV/r/+DSV±RBV) Therapy in Recurrent Hepatitis C Virus (HCV) Genotype 1 Infection Post-Liver Transplant: AMBER-CEE Study. Ann. Transpl..

[31] Ma Z., de Man R.A., Kamar N., Pan Q. (2022). Chronic hepatitis E: Advancing research and patient care. J. Hepatol..

[32] Primadharsini P.P., Nagashima S., Nishiyama T., Takahashi M., Murata K., Okamoto H. (2022). Development of Recombinant Infectious Hepatitis E Virus Harboring the nanoKAZ Gene and Its Application in Drug Screening. J. Virol..

[33] Primadharsini P.P., Nagashima S., Takahashi M., Murata K., Okamoto H. (2022). Ritonavir Blocks Hepatitis E Virus Internalization and Clears Hepatitis E Virus In Vitro with Ribavirin. Viruses.

[34] Pereira-Chioccola V.L., Vidal J.E., Su C. (2009). Toxoplasma gondii infection and cerebral toxoplasmosis in HIV-infected patients. Future Microbiol..

[35] Abou-El-Naga I.F., El Kerdany E.D., Mady R.F., Shalaby T.I., Zaytoun E.M. (2017). The effect of lopinavir/ritonavir and lopinavir/ritonavir loaded PLGA nanoparticles on experimental toxoplasmosis. Parasitol. Int..

[36] Beld L., Jung H., Bulman C.A., Rosa B.A., Fischer P.U., Janetka J.W., Lustigman S., Sakanari J.A., Mitreva M. (2022). Aspartyl Protease Inhibitors as Anti-Filarial Drugs. Pathogens.

[37] Alves É.A.R., de Miranda M.G., Borges T.K., Magalhães K.G., Muniz-Junqueira M.I. (2015). Anti-HIV drugs, lopinavir/ritonavir and atazanavir, modulate innate immune response triggered by Leishmania in macrophages: The role of NF-κB and PPAR-γ. Int. Immunopharmacol..

[38] Brilhante R.S., Caetano É.P., Riello G.B., Guedes G.M., Castelo-Branco Dde S., Fechine M.A., Oliveira J.S., Camargo Z.P., Mesquita J.R., Monteiro A.J. (2016). Antiretroviral drugs saquinavir and ritonavir reduce inhibitory concentration values of itraconazole against Histoplasma capsulatum strains in vitro. Braz. J. Infect. Dis..

[39] van der Putten E., Wosikowski K., Beijnen J.H., Imre G., Freund C.R. (2024). Ritonavir reverses resistance to docetaxel and cabazitaxel in prostate cancer cells with acquired resistance to docetaxel. Cancer Drug Resist..

[40] Ikezoe T., Hisatake Y., Takeuchi T., Ohtsuki Y., Yang Y., Said J.W., Taguchi H., Koeffler H.P. (2004). HIV-1 protease inhibitor, ritonavir: A potent inhibitor of CYP3A4, enhanced the anticancer effects of docetaxel in androgen-independent prostate cancer cells in vitro and in vivo. Cancer Res..

[41] Staal J., Beyaert R. (2018). Inflammation and NF-κB Signaling in Prostate Cancer: Mechanisms and Clinical Implications. Cells.

[42] Skinner K.T., Palkar A.M., Hong A.L. (2023). Genetics of ABCB1 in Cancer. Cancers.

[43] Lima T.S., Souza L.O., Iglesias-Gato D., Elversang J., Jørgensen F.S., Kallunki T., Røder M.A., Brasso K., Moreira J.M.A. (2022). Itraconazole Reverts ABCB1-Mediated Docetaxel Resistance in Prostate Cancer. Front. Pharmacol..

[44] Mita A.C., Figlin R., Mita M.M. (2012). Cabazitaxel: More than a new taxane for metastatic castrate-resistant prostate cancer?. Clin. Cancer Res..

[45] de Weger V.A., Stuurman F.E., Hendrikx J.J.M.A., Moes J.J., Sawicki E., Huitema A.D.R., Nuijen B., Thijssen B., Rosing H., Keessen M. (2017). A dose-escalation study of bi-daily once weekly oral docetaxel either as ModraDoc001 or ModraDoc006 combined with ritonavir. Eur. J. Cancer.

[46] Vaishampayan U.N., Keessen M., Heath E.I., Dreicer R., Buchler T., Árkosy P.F., Csoszi T., Wiechno P.J., Kholtobin D., Kopyltsov E. (2022). A phase 2 randomized study of oral docetaxel plus ritonavir (ModraDoc006/r) in patients with metastatic castration-resistant prostate cancer (mCRPC). J. Clin. Oncol..

[47] Li X., Ng A.S.N., Mak V.C.Y., Chan K.K.L., Cheung A.N.Y., Cheung L.W.T. (2020). Strategic Combination Therapies for Ovarian Cancer. Curr. Cancer Drug Targets.

[48] Kumar S., Bryant C.S., Chamala S., Qazi A., Seward S., Pal J., Steffes C.P., Weaver D.W., Morris R., Malone J.M. (2009). Ritonavir blocks AKT signaling, activates apoptosis and inhibits migration and invasion in ovarian cancer cells. Mol. Cancer.

[49] Vélez-Cruz R., Johnson D.G. (2017). The Retinoblastoma (RB) Tumor Suppressor: Pushing Back against Genome Instability on Multiple Fronts. Int. J. Mol. Sci..

[50] Narasimha A.M., Kaulich M., Shapiro G.S., Choi Y.J., Sicinski P., Dowdy S.F. (2014). Cyclin D activates the Rb tumor suppressor by mono-phosphorylation. eLife.

[51] Winterhoff B., Teoman A., Freyer L., Von Bismarck A., Dowdy S., Schmalfeldt B., Kumar S., Shridhar V. (2013). The HIV protease inhibitor ritonavir induces cell cycle arrest and apoptosis in the A2780 ovarian cancer cell line in vitro and in vivo. Gynecol. Oncol..

[52] Rascio F., Spadaccino F., Rocchetti M.T., Castellano G., Stallone G., Netti G.S., Ranieri E. (2021). The Pathogenic Role of PI3K/AKT Pathway in Cancer Onset and Drug Resistance: An Updated Review. Cancers.

[53] Momenimovahed Z., Tiznobaik A., Taheri S., Salehiniya H. (2019). Ovarian cancer in the world: Epidemiology and risk factors. Int. J. Womens Health.

[54] Srirangam A., Wang M., Blum J., Einhorn L., Potter D.A. (2005). Ritonavir causes G1 arrest in non-small cell lung cancer (NSCLC), in part, by binding hsp90 and down-regulating Cdk4 and other Hsp90 client proteins. Cancer Res..

[55] Rong B., Yang S. (2018). Molecular mechanism and targeted therapy of Hsp90 involved in lung cancer: New discoveries and developments (Review). Int. J. Oncol..

[56] Srirangam A., Milani M., Mitra R., Guo Z., Rodriguez M., Kathuria H., Fukuda S., Rizzardi A., Schmechel S., Skalnik D.G. (2011). The Human Immunodeficiency Virus Protease Inhibitor Ritonavir Inhibits Lung Cancer Cells, in Part, by Inhibition of Survivin. J. Thorac. Oncol..

[57] Li D., Hu C., Li H. (2018). Survivin as a novel target protein for reducing the proliferation of cancer cells. Biomed. Rep..

[58] Arora L., Kumar A.P., Arfuso F., Chng W.J., Sethi G. (2018). The Role of Signal Transducer and Activator of Transcription 3 (STAT3) and Its Targeted Inhibition in Hematological Malignancies. Cancers.

[59] Courtney D., Davey M.G., Moloney B.M., Barry M.K., Sweeney K., McLaughlin R.P., Malone C.M., Lowery A.J., Kerin M.J. (2022). Breast cancer recurrence: Factors impacting occurrence and survival. Ir. J. Med. Sci..

[60] Srirangam A., Mitra R., Wang M., Gorski J.C., Badve S., Baldridge L., Hamilton J., Kishimoto H., Hawes J., Li L. (2006). Effects of HIV protease inhibitor ritonavir on Akt-regulated cell proliferation in breast cancer. Clin. Cancer Res..

[61] Li H., Prever L., Hirsch E., Gulluni F. (2021). Targeting PI3K/AKT/mTOR Signaling Pathway in Breast Cancer. Cancers.

[62] Liu Y., Ma H., Yao J. (2020). ERα, A Key Target for Cancer Therapy: A Review. Onco Targets Ther..

[63] Yu H., Hendrikx J.J., Rottenberg S., Schellens J.H., Beijnen J.H., Huitema A.D. (2016). Development of a Tumour Growth Inhibition Model to Elucidate the Effects of Ritonavir on Intratumoural Metabolism and Anti-tumour Effect of Docetaxel in a Mouse Model for Hereditary Breast Cancer. AAPS J..

[64] Schellens J.H., Malingré M.M., Kruijtzer C.M., Bardelmeijer H.A., van Tellingen O., Schinkel A.H., Beijnen J.H. (2000). Modulation of oral bioavailability of anticancer drugs: From mouse to man. Eur. J. Pharm. Sci..

[65] Hendrikx J.J., Lagas J.S., Song J.Y., Rosing H., Schellens J.H., Beijnen J.H., Rottenberg S., Schinkel A.H. (2016). Ritonavir inhibits intratumoral docetaxel metabolism and enhances docetaxel antitumor activity in an immunocompetent mouse breast cancer model. Int. J. Cancer.

[66] Gote V., Sharma A.D., Pal D. (2021). Hyaluronic Acid-Targeted Stimuli-Sensitive Nanomicelles Co-Encapsulating Paclitaxel and Ritonavir to Overcome Multi-Drug Resistance in Metastatic Breast Cancer and Triple-Negative Breast Cancer Cells. Int. J. Mol. Sci..

[67] Asano M., Tanaka S., Sakaguchi M. (2020). Effects of normothermic microwave irradiation on CD44(+)/CD24(−) in breast cancer MDA-MB-231 and MCF-7 cell lines. Biosci. Biotechnol. Biochem..

[68] Al-Othman N., Alhendi A., Ihbaisha M., Barahmeh M., Alqaraleh M., Al-Momany B.Z. (2020). Role of CD44 in breast cancer. Breast Dis..

[69] Snyder S., Murundi S., Crawford L., Putnam D. (2020). Enabling P-glycoprotein inhibition in multidrug resistant cancer through the reverse targeting of a quinidine-PEG conjugate. J. Control. Release.

[70] Tabas I., Ron D. (2011). Integrating the mechanisms of apoptosis induced by endoplasmic reticulum stress. Nat. Cell Biol..

[71] Sato A. (2015). The human immunodeficiency virus protease inhibitor ritonavir is potentially active against urological malignancies. Onco Targets Ther..

[72] Gaedicke S., Firat-Geier E., Constantiniu O., Lucchiari-Hartz M., Freudenberg M., Galanos C., Niedermann G. (2002). Antitumor effect of the human immunodeficiency virus protease inhibitor ritonavir: Induction of tumor-cell apoptosis associated with perturbation of proteasomal proteolysis. Cancer Res..

[73] Sato A., Asano T., Okubo K., Isono M., Asano T. (2017). Ritonavir and ixazomib kill bladder cancer cells by causing ubiquitinated protein accumulation. Cancer Sci..

[74] Xie J., Wan N., Liang Z., Zhang T., Jiang J. (2019). Ixazomib—The first oral proteasome inhibitor. Leuk. Lymphoma.

[75] Chen X., Shi C., He M., Xiong S., Xia X. (2023). Endoplasmic reticulum stress: Molecular mechanism and therapeutic targets. Signal Transduct. Target. Ther..

[76] Sato A., Asano T., Okubo K., Isono M., Asano T. (2018). Nelfinavir and Ritonavir Kill Bladder Cancer Cells Synergistically by Inducing Endoplasmic Reticulum Stress. Oncol. Res..

[77] Okubo K., Isono M., Asano T., Sato A. (2019). Lopinavir-Ritonavir Combination Induces Endoplasmic Reticulum Stress and Kills Urological Cancer Cells. Anticancer. Res..

[78] Okubo K., ReßIng N., Schulz W.A., Hansen F.K., Asano T., Sato A. (2021). The Dual Histone Deacetylase-Proteasome Inhibitor RTS-V5 Acts Synergistically With Ritonavir to Induce Endoplasmic Reticulum Stress in Bladder Cancer Cells. Anticancer. Res..

[79] Batchu R.B., Gruzdyn O.V., Bryant C.S., Qazi A.M., Kumar S., Chamala S., Kung S.T., Sanka R.S., Puttagunta U.S., Weaver D.W. (2014). Ritonavir-Mediated Induction of Apoptosis in Pancreatic Cancer Occurs via the RB/E2F-1 and AKT Pathways. Pharmaceuticals.

[80] Mandigo A.C., Yuan W., Xu K., Gallagher P., Pang A., Guan Y.F., Shafi A.A., Thangavel C., Sheehan B., Bogdan D. (2021). RB/E2F1 as a Master Regulator of Cancer Cell Metabolism in Advanced Disease. Cancer Discov..

[81] Zhao L., Zhao Y., Schwarz B., Mysliwietz J., Hartig R., Camaj P., Bao Q., Jauch K.W., Guba M., Ellwart J.W. (2016). Verapamil inhibits tumor progression of chemotherapy-resistant pancreatic cancer side population cells. Int. J. Oncol..

[82] Pontious C., Kaul S., Hong M., Hart P.A., Krishna S.G., Lara L.F., Conwell D.L., Cruz-Monserrate Z. (2019). Cathepsin E expression and activity: Role in the detection and treatment of pancreatic cancer. Pancreatology.

[83] Keliher E.J., Reiner T., Earley S., Klubnick J., Tassa C., Lee A.J., Ramaswamy S., Bardeesy N., Hanahan D., Depinho R.A. (2013). Targeting cathepsin E in pancreatic cancer by a small molecule allows in vivo detection. Neoplasia.

[84] Spencer N.Y., Stanton R.C. (2019). The Warburg Effect, Lactate, and Nearly a Century of Trying to Cure Cancer. Semin. Nephrol..

[85] McBrayer S.K., Cheng J.C., Singhal S., Krett N.L., Rosen S.T., Shanmugam M. (2012). Multiple myeloma exhibits novel dependence on GLUT4, GLUT8, and GLUT11: Implications for glucose transporter-directed therapy. Blood.

[86] Weir P., Donaldson D., McMullin M.F., Crawford L. (2023). Metabolic Alterations in Multiple Myeloma: From Oncogenesis to Proteasome Inhibitor Resistance. Cancers.

[87] Dalva-Aydemir S., Bajpai R., Martinez M., Adekola K.U., Kandela I., Wei C., Singhal S., Koblinski J.E., Raje N.S., Rosen S.T. (2015). Targeting the metabolic plasticity of multiple myeloma with FDA-approved ritonavir and metformin. Clin. Cancer Res..

[88] Schaff L.R., Mellinghoff I.K. (2023). Glioblastoma and Other Primary Brain Malignancies in Adults: A Review. JAMA.

[89] Kast R.E., Ramiro S., Lladó S., Toro S., Coveñas R., Muñoz M. (2016). Antitumor action of temozolomide, ritonavir and aprepitant against human glioma cells. J. Neurooncol.

[90] Rauschenbach L., Wieland A., Reinartz R., Kebir S., Till A., Darkwah Oppong M., Dobersalske C., Ullrich V., Ahmad A., Jabbarli R. (2020). Drug repositioning of antiretroviral ritonavir for combinatorial therapy in glioblastoma. Eur. J. Cancer.

[91] Gratton R., Tricarico P.M., Guimaraes R.L., Celsi F., Crovella S. (2018). Lopinavir/Ritonavir Treatment Induces Oxidative Stress and Caspaseindependent Apoptosis in Human Glioblastoma U-87 MG Cell Line. Curr. HIV Res..

[92] Hsu A., Granneman G.R., Bertz R.J. (1998). Ritonavir: Clinical pharmacokinetics and interactions with other anti-HIV agents. Clin. Pharmacokinet..

